# Oncological Analysis and Surgical Outcomes in Postcricoid Carcinoma: A 14 Years Retrospective Study

**DOI:** 10.3390/cancers14133146

**Published:** 2022-06-27

**Authors:** Chun Chen, Lei Hang, Yupeng Liu, Jin Xie, Jun Yang

**Affiliations:** 1 Department of Otorhinolaryngology-Head & Neck Surgery, Xinhua Hospital, Shanghai Jiao Tong University School of Medicine, Shanghai 200092, China; acbbaz@sjtu.edu.cn (C.C.); liuyupeng@xinhuamed.com.cn (Y.L.); 2 Ear Institute, Shanghai Jiao Tong University School of Medicine, Shanghai 200092, China; 3 Shanghai Key Laboratory of Translational Medicine on Ear and Nose Diseases, Shanghai 200092, China; 4 Business School, Shanghai Normal University Tianhua College, Shanghai 201815, China; hanglei@jejunu.ac.kr

**Keywords:** postcricoid carcinoma, laryngeal function, recurrence, survival rate, prognosis

## Abstract

**Simple Summary:**

High local failure and treatment-related morbidity represent a great challenge for postcricoid carcinoma which appears to be more aggressive and requires more morbid oncologic surgery with reconstruction of the larynx and hypopharynx. This paper presents a retrospective analysis focusing on the evaluation of clinical characteristics, therapeutic approaches, quality of swallowing, voice rehabilitation, and verbal communication after a long-term follow-up. To date, this is the first and largest cohort published to reveal a deep understanding of clinical routines in postcricoid carcinoma.

**Abstract:**

Background: Postcricoid carcinoma is a rare but aggressive type of hypopharyngeal carcinoma with poor prognosis and high mortality; thus, it is indispensable to investigate the surgical efficacy and multimodal strategies. Methods: This retrospective study included postcricoid carcinoma patients undergoing surgical resection from 2008 to 2022. Treatment methods and clinical characteristics were analyzed to evaluate prognostic factors for oncological outcomes. Results: Of 72 patients, 13 cases were in the I–II stage and 59 in the III–IV stage. The overall survival (OS) was 50.0%; the laryngeal function preservation rate was 69.4%. Univariate analysis found that high mortality was associated with low tumor differentiation, lymph node metastasis, neck recurrence, and smoke history via log-rank test (*p* < 0.05); postoperative radiotherapy (RT) remained positive in OS (*p* = 0.04). The multivariable model further revealed that lymph node metastasis was a dominant determinant after accounting for covariates (HR 1.75; 95% CI 0.85–3.59). The data also indicated that neoadjuvant chemotherapy (NAC) and tumor diameter ≤ 2 cm were causing lower rates of pharyngeal fistula and locoregional relapse. Conclusions: Surgeons should emphasize high-risk features and optimize individualized surgical procedures for postcricoid carcinoma patients. Combined with multimodal treatments, it is feasible to reconstruct laryngeal function and lessen postoperative morbidities in advanced patients.

## 1. Introduction

The postcricoid region is located behind the cricoarytenoid joint and the backside of the cricoid cartilage, connected to the esophagus at the lower edge [1]. The number of primary tumors from the postcricoid region is the least versus the other two types of hypopharyngeal carcinoma (pyriform sinus and posterior wall), with an incidence rate of about 5% [2]. Dining habits contribute to high incidence rates in China, however, the 5-year survival rate of advanced hypopharyngeal carcinoma was reported to be only 30–35% with comprehensive therapy [3,4]. In light of the anatomical location, postcricoid carcinoma is often diagnosed in an advanced stage or after peripheral invasion, increasing difficulties in the reconstruction of the functional pharyngoesophageal segment [5]. Furthermore, lymph node metastasis is often related to advanced local disease, combined with extracapsular extension or positive margins, which has been demonstrated as the most pivotal factor of survival in head and neck carcinoma [6]. As is often the case, the incidence of distant metastasis is up to 60% at initial diagnosis, postcricoid carcinoma is recognized as one of the most refractory head and neck malignant tumors [7].

Previous work remains narrow in consensus regarding surgical methods and oncologic outcomes of postcricoid carcinoma, far exceeding the challenges of other types. Operation (OP) is considered a standard remedy for postcricoid carcinoma, while recent studies promote multimodal strategies, including radiotherapy (RT), neoadjuvant chemotherapy (NAC), concurrent chemoradiation (CRT), and immunotherapy [8,9]. Pessimistically, quantities of cases developed locoregional relapse and neck recurrence after primary laryngectomy or definitive nonsurgical treatments, with an unsatisfactory quality of life. Thus, a small patient population tends to choose nonsurgical treatment or even palliative care to avoid the permanent sacrifice of laryngeal function and postoperative morbidities, which may influence the surgical choice that favors survival [3].

On the other hand, despite the progress in the latest therapeutic techniques, the quantity of survival should not be the only endpoint, postoperative events such as locoregional relapse, distant metastases, and severe morbidities are important outcomes as well. It is intuitive that the reconstructive of airways, phonatory, and swallowing tubes requires well-experienced techniques and individual regimens, or the patients’ appearance and social adaptability would be affected directly [10].

In this study, a total of 72 cases were investigated, which, to our knowledge, is the most significant cohort analysis in published research. This study first examines the relevance of various clinical characteristics and surgical outcomes, as well as analyses regarding pharyngeal fistula, decannulation rate, swallowing, voice, and respiratory function, for further comparing the utility of multimodal regimens and refining organ-preservation surgical protocols.

## 2. Materials and Methods

### 2.1. Patients and Therapeutic Strategies

**Figure 1:**
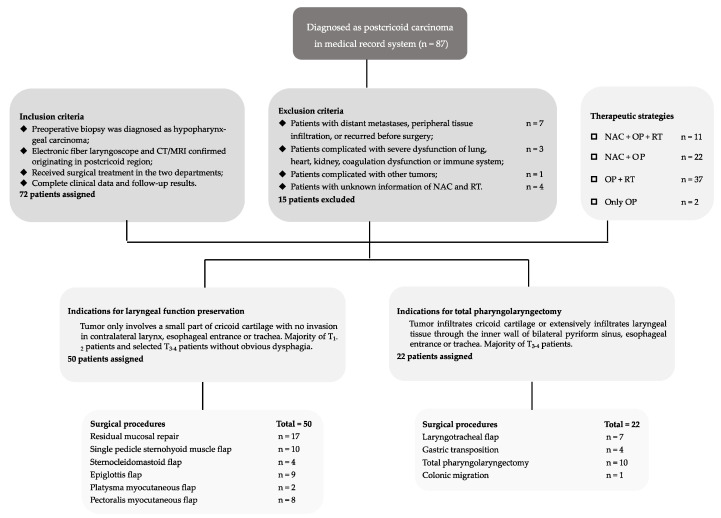
Flow of retrospective patients.

Between January 2008 and March 2022, 72 cases of postcricoid carcinoma met inclusion criteria in the department of otorhinolaryngology-head and neck surgery, Shanghai General Hospital, and Xinhua Hospital for follow-up surveys (ethics code: XHEC-WJW-2020-032). Queried patients underwent surgical resections by the same senior surgeon team, while NAC and RT were determined based on their own aspirations and economic conditions. Surgical procedures were designed by best indicator regarding tumor extent, lymph nodes invasion, and general status. The detailed screening process is shown in the flow (Figure 1).

**Figure 2:**
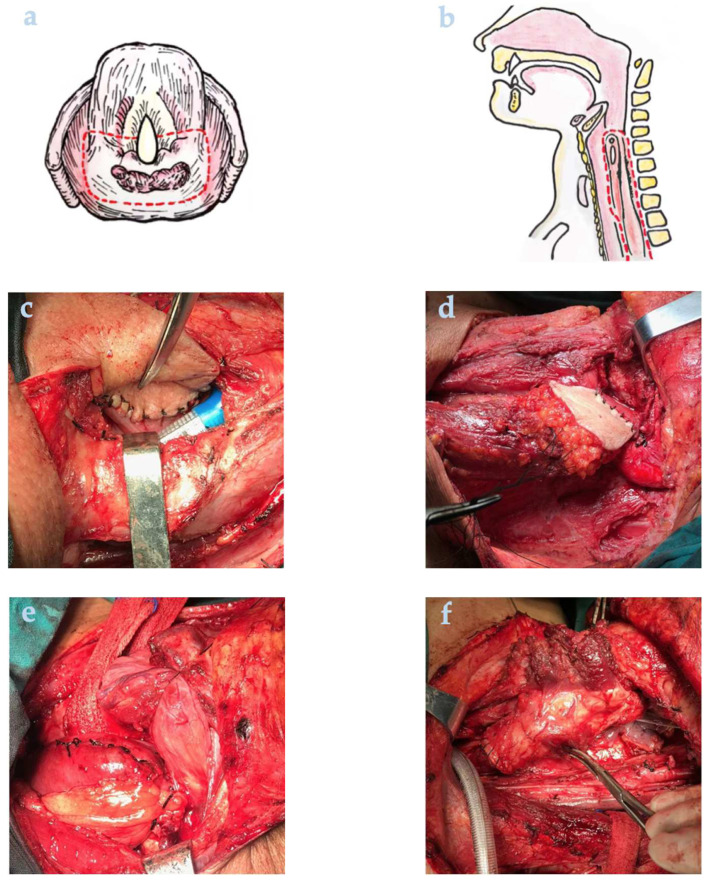
Illustrations of surgical procedures. (**a**) Resection extent of early stage cases (T_1–2_ postcricoid carcinoma). (**b**) Resection extent of advanced stage cases (T_3–4_ postcricoid carcinoma). (**c**) Modified platysma myocutaneous flap repaired the lateral posterior wall of the hypopharynx. (**d**) Pectoralis myocutaneous flap repaired posterolateral defect. (**e**) Gastric transposition (laryngeal function not preserved). (**f**) Laryngotracheal flap restored laryngeal and hypopharyngeal defects.

Standard radical resection was based on National Comprehensive Therapy Network (NCCN) guidelines [11]. Neck dissection extent was determined by tumor location with adequate guidance of enhanced CT/MRI. The primaries were removed by a safety margin >1 cm (Figure 2a,b). Management of repairing methods varied with the defect ranges. If the anterior wall defected, the postcricoid area could be sutured by the mucosa from the inner wall of the pyriform sinus and retaining mucosa. If the posterior wall defected, the outer wall of the pyriform sinus could be used to restore the postcricoid region. The posterolateral defect could be restored with a sternocleidomastoid flap or pectoralis myocutaneous flap (Figure 2c,d). If arytenoid cartilage or cricoid cartilage is infiltrated, a single pedicle sternohyoid muscle flap or epiglottis flap could be used for reconstruction. When the tumor invaded the esophagus, it was well worth considering whether to preserve laryngeal function according to the invasion of the larynx. Gastric transposition was performed with general surgeons (Figure 2e). Application of laryngotracheal flap was a method of not laryngeal preservation type (Figure 2f). Total laryngeal hypopharyngectomy was the most used, especially in those aged, with poor performance status and very advanced stage patients.

The NAC regimen consisted of intravenous infusions of 75 mg/m^2^ docetaxel plus cisplatin 100 mg/m^2^ plus 5-fluorouracil 1000 mg/m^2^/day (TPF regimen) [12,13]. Every 3 weeks served as a cycle; after 2 cycles, they received electronic laryngoscopy and imaging examination to evaluate the efficacy. Then we developed an individualized program for surgical treatment. Postoperative adjuvant radiotherapy techniques were performed with intensity-modulated radiotherapy (IMRT). The radiotherapy dose was 50–70 Gy, and the radiotherapy range included the tumor bed and high-risk subclinical areas involved extracapsular extension or surgical margins nearby (2.0 Gy per fraction, 5 fractions per week). All RT and NAC cases were treated in our departments.

### 2.2. Statistical Analysis

Measure data were described as mean ± standard deviation (SD). Qualitative parameters were described as frequency or percentage. Univariate and multivariate analyses were performed to detect the connection between independent risk factors of prognosis using R (version 3.6.2) with “Survival” packages. R software also realized Kaplan–Meier curves, log-rank tests, and forest plots. Results were shown as hazard ratio (HR) with 95% confidence intervals (CI). The chi-square test was performed using IBM-SPSS version 25.0 to evaluate the treatment effects with relevant factors of surgical treatment. We repeated all experiments at least three times. A *p*-value < 0.05 was considered statistically significant.

## 3. Results

### 3.1. Patient Characteristics

The follow-up time ranged from 5 to 160 months, with an average of 24.75 months. This cohort had a strong male preponderance (male:female, 68:4). The mean age was 60.22 (39–77 years, SD 7.12). The first clinical symptoms included 34 (47.2%) cases of pharyngeal foreign body sensation, 17 (23.6%) cases of s dysphagia, 7 (9.7%) cases of hoarseness, and 14 (19.4%) cases of cervical nodules. Pharyngeal foreign body sensation was the most prominent symptom in the early stage, while the typical symptoms of advanced patients involved dysphagia, hoarseness, dyspnea, and cervical nodules.

As for NAC outcomes, 17 (51.5%) cases achieved partial remission (PR), 12 (36.4%) cases were stable (SD) and 4 (12.1%) cases were progressive (PD). Of the 72 patients, 50 (69.4%) patients received partial or total laryngeal function preservation, and 22 (30.6%) patients lost laryngeal function. The larynx preservation rate was 69.7% (23/33) in the NAC group. All cN_+_ patients were suggested to undergo postoperative radiotherapy, but 3 patients declined further intervention. Table 1 shows the TNM stage classification according to the 8th edition of the Union for International Cancer Control/American Joint Committee on Cancer (UICC/AJCC). There were 8 cases in stage I, 5 cases in stage II, 9 cases in stage III, and 50 cases in stage IV.

### 3.2. Survival Analysis

In the entire cohort, the overall survival rate (OS) was 50%, while the OS was 61.1% at 36 months and 34.4% at 60 months. The baseline characteristics are shown in Table 2. The findings comprised 39 (54.2%) locoregional relapse, 37 (51.4%) neck recurrence cases. Among the 36 deaths, 14 cases died of relapse in primary; 12 cases died of neck recurrence with carotid artery invasion; 2 cases died of heart failure during hospitalization; 3 cases died of acute myocardial infarction after salvage surgery; 1 case died of postoperative pulmonary infection; 1 case died of pulmonary embolism; 3 cases died of distant metastasis (2 cases died of esophagus and 1 case died of pulmonary metastasis).

**Figure 3:**
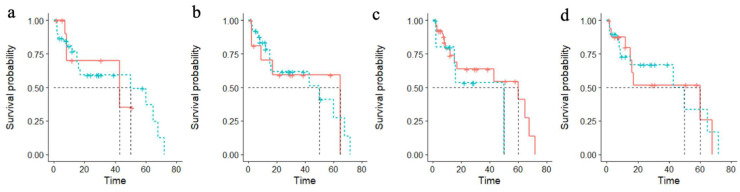

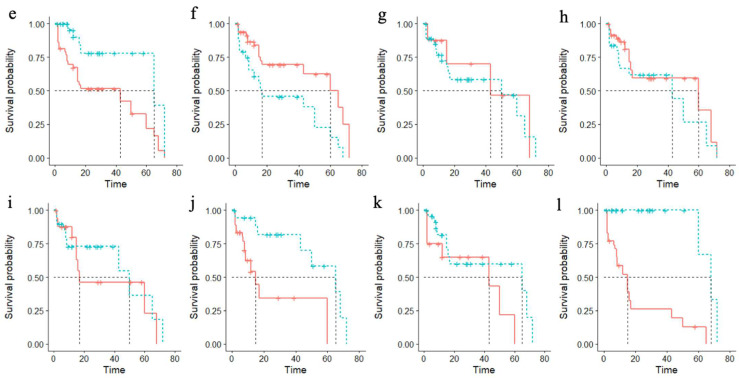
Comparison of Kaplan–Meier survival curves in different treatment and clinical characteristics. (**a**) Green: TNM III–IV stage; red: TNM I–II stage; (**b**) green: laryngeal function preserved; red: laryngeal function not preserved; (**c**) green: elective neck dissection; red: radical neck dissection; (**d**) green: no NAC; red: NAC; (**e**) green: high/medium differentiated tumor; red: low differentiated tumor; (**f**) green: cN_+_; red: cN_0_; (**g**) green: tumor size > 2 cm; red: tumor size ≤ 2 cm; (**h**) green: pharyngeal fistula; red: no pharyngeal fistula; (**i**) green: recurrence in situ; red: no recurrence in situ; (**j**) green: no smoke history; red: smoke history; (**k**) green: RT group; red: no RT group; (**l**) green: no neck recurrence; red: neck recurrence.

Univariate factor analysis of risk factors by Kaplan–Meier survival curves is shown in Figure 3. In addition to matched variables, no significant differences were observed in OS between I–II stage patients versus III–IV stage patients (38.6% versus 47.5%, *p* = 0.85) and in patients with a tumor diameter ≤ 2 cm versus > 2 cm (50.0% versus 50.0%, *p* = 0.63). Despite this, patients with cN+ showed significant in increased mortality versus cN0 cases (62.5% versus 25.0%, *p* = 0.01). Not surprisingly, we found the concordance between neck metastasis and neck recurrence on the influence of OS (*p* < 0.001).

Regarding the resection methods of tumor and lymph node metastasis, there was no significant difference in laryngx preservation or neck dissection extent (*p* = 0.83, *p* = 0.22). The mean tumor diameter was 3.08 cm (ranged 0.8–6.5 cm). All cases were diagnosed with squamous cell carcinoma by histopathology, 29 (40.3%) cases of high/medium tumor differentiation, and 43 (59.7%) cases of low degree. Pathological evidence showed that the number of low differentiation patients was significantly more than those with high/medium differentiation, additionally, 30/43 (69.8%) of low differentiation patients died of the primary. Meanwhile, postoperative RT was associated with improved survival compared with those without further intervention (*p* < 0.05), while NAC was not associated with OS (*p* = 0.81). Tobacco consumption was confirmed as a major predictor of postcricoid carcinoma; 36 (50.0%) patients had a smoke history in this cohort (*p* < 0.001). On average, 16 (44.4%) of them were heavy smokers (>15 cigarettes/day), 7 (19.4%) of them were moderate smokers (6–15 cigarettes/day) and 13 (36.2%) of them were light smokers (<5 cigarettes/day) in the last decade.

Variables correlated with survival built a multivariate regression model (Table 3). Cervical lymph node metastasis was clarified as a strong risk factor for increased mortality in postcricoid carcinoma patients (HR 1.75; 95% CI 0.85–3.59).

### 3.3. Surgical Treatment Efficacy

#### 3.3.1. Swallowing Function

After nasal feeding, the nasogastric tube was removed in 14 to 33 days, with a mean of 21.7 days. In general, 48 (66.7%) cases were free of nasopharyngeal reflux, intaking foods with no restrictions but pharyngeal foreign body sensation; 10 cases had mild nasopharyngeal reflux, who overcame it through oral intake of food and liquid in one week spontaneously. One case of severe nasopharyngeal reflux developed dysphagia after RT, depending on prolonged parenteral feeding at last. Anastomotic strictures occurred in two cases restored by pectoralis myocutaneous flap and an esophageal stent was placed under general anesthesia.

As to perioperative pharyngeal fistula, 25 (34.7%) patients suffered pharyngeal fistula, 16 (64.0%) of whom retained laryngeal function, and 9 (36.0%) of whom did not. There were 23 (92.0%) cases of infectious pharyngeal fistula and 2 (8.0%) cases of chylous fistula. The occurrence time ranged from 6 to 28 days, the mean time was 12.7 days. The pharyngeal fistula was cured in 7 to 36 days, with an average of 14.5 days. Among them, 22 cases were cured after dressing change; 2 cases were performed repair operation with pectoralis myocutaneous flap after the first operation failed, without deep space infections or abscesses observed at the donor site. One case refused salvage operation and underwent gastrostomy in a local hospital instead, died of lung infection later. We investigated the relationship between clinical characteristics and pharyngeal fistula. It was found that NAC and tumor diameter ≤ 2 cm size were protective factors of pharyngeal fistula (*p* < 0.001; *p* = 0.03), whereas larynx preservation, RT, TNM stage, and neck dissection extent were not statistically associated with the incidence of pharyngeal fistula (*p* > 0.05) (Table 4).

#### 3.3.2. Voice Function

**Figure 4:**
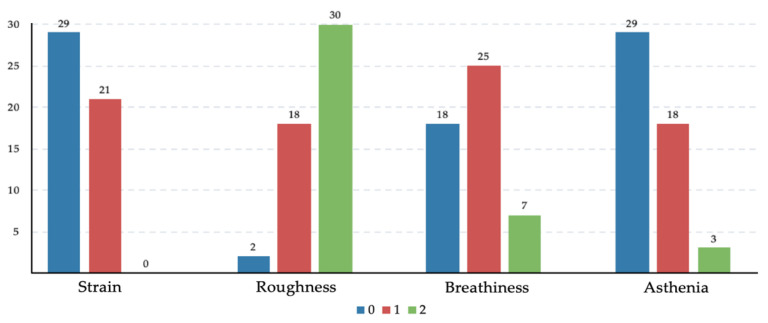
Histograms of GRABS scale after decannulation. Blue presents normal (grade 0), red presents slight (grade 1), green presents moderate (grade 2).

Of the 50 cases in the laryngeal function preservation group, 31 (62.0%) cases had a clear articulation, while 19 (38.0%) cases had mild dysphonia after reconstruction, all of whom performed an understandable voice. For patients who did not retain laryngeal function (*n =* 22), 4 (18.2%) patients were accustomed to electronic larynx skillfully, whereas 2 patients attempted to use it but ultimately failed. None of the remaining patients alive made a sound. Subjective auditory assessments followed the GRABS (Grade, Strain, Roughness, Breathiness, Asthenia) scale developed by the Japanese Society for Logopedics and Phoniatrics [14]. The voices were scored by a specialist otorhinolaryngologist in a quiet outpatient clinic or by phone. Notably, a majority of cases had rough pronunciation, the moderate level in roughness was 60.0%, and the slight level in strain, breathiness, and asthenia was 42.0%, 50.0%, and 36% relatively, which suggested that hoarseness and slight polypnea developed frequently probably because of the scar proliferation and anastomotic stenosis of the new larynx after reconstruction (Figure 4).

#### 3.3.3. Respiratory Function

Of the 50 laryngeal function preservation cases, 18 (36.0%) patients removed the tracheal tube; the decannulation time ranged from 89 to 375 days, with a mean duration of 172.5 days. Furthermore, 12 (24.0%) cases could be tube-blocked intermittently; nonetheless, their daily activities and nighttime sleep were interrupted, so they failed in decannulation. Four cases were decannulated and recurred in primary at 3, 7, 9, and 36 months respectively, then underwent salvage total laryngectomy (STL) and tracheostomy again. Decannulation and possible related factors were assessed by the chi-square test (Table 5). A statistically low decannulation rate was found in irradiated patients, which may be related to the side effects as keloids or tissue atrophy after RT (*p* = 0.02).

#### 3.3.4. Locoregional Relapse and Neck Metastasis

As in the survival analysis, locoregional relapse showed no significant correlation with OS (*p* = 0.37). There were 39 (54.2%) cases that recurred in situ after the first operation (Table 6); 27 (69.2%) cases retained laryngeal function, 12 (30.8%) cases did not (*p* = 0.97). The recurrence time ranged from 1 to 47 months, with an average of 18.7 months; the 3- and 5-year disease-free survival (DFS) rates were 71.9% and 35.7%. TNM stage was not significantly associated with locoregional relapse (*p* > 0.05), in particular, 33 (84.6%) locoregional relapse patients were in III–IV tumor stages. Moreover, 14 (87.5%) tumor diameter ≤ 2 cm patients have not developed locoregional relapse so far (*p* < 0.001). The chi-square test showed that RT did not significantly affect the locoregional relapse rate (*p* = 0.32); however, the rate was dramatically lower in the NAC group than that in the no NAC group (*p* < 0.001).

Eight cases relapsed both in situ and lymph nodes. One case recurred at the tracheostomy site. In this case, the primary tumor recurred in the first 4 months after the primary was removed. The tracheostomy site recurrence was found 3 months after STL and returned to the local hospital for palliative radiotherapy.

If the primary was located closed to the midline, contralateral neck dissection was necessary, 26 (36.1%) patients underwent bilateral neck dissection. Based on the size of the metastatic lymph nodes and local invasion, 21 (29.2%) patients underwent radical neck dissection, and 51 (70.8%) patients underwent elective dissection. Postoperative pathology reported lymph node metastasis in 48 cases. The median number of positive nodes ranged from 4 to 45, with a mean number of 11.4; 27 (37.5%) patients suffered from lymphovascular and extracapsular invasion. The relapse time of lymph nodes ranged from 3 to 52 months, with an average of 12.1 months. A total of 24 patients recurred on the affected side, 7 on the contralateral side, and 6 on both sides with extracapsular invasion. Among them, three patients lost the secondary surgical chance due to carotid artery invasion. The rest underwent radical neck dissection, diagnosed with metastatic carcinoma by pathology. The chi-square test showed that neck recurrence had no significant correlation between NAC, RT, TNM stage, and tumor size (*p* > 0.05) (Table 7). Furthermore, cN_0_ had a potential application in recurrent assessment (*p* = 0.03), while the resection methods of nodal metastases were not significantly associated with the recurrence rate (radical versus elective: 47.6% versus 52.9%, *p* = 0.68).

## 4. Discussion

This study first provides insights into the clinical features, larynx preservation protocols, multimodal strategies, and postoperative morbidities of postpricoid carcinoma. An exception is the publication by Wei et al., they described a total of 45 postcricoid carcinoma cases, including 1 case in stage I, 4 cases in stage II, 10 cases in stage III, and 30 cases in stage IV, with a positive rate of cervical metastasis 73.3% (33/45), which echoed the same findings that postcricoid carcinoma is an aggressive subtype of hypopharyngeal carcinoma [10]. Therefore, it is important to sum up experience from limited case resources.

Postcricoid region tumor frequently invades cricoid cartilage, then thyroid, trachea, and recurrent laryngeal nerve, leading to the palsy of unilateral even bilateral vocal cord [15]. Because of the local infiltration, most postcricoid carcinomas were in an advanced stage when clinically diagnosed in this study. The oncological outcomes were relatively satisfactory; the 3- and 5-year DFS and OS were 71.9%, 35.7%, and 61.1%, 34.4%. Cervical lymph node metastasis and recurrence are predicted strongly for survival. Meanwhile, smoking history represented a high-risk factor for mortality.

Recently, multimodal treatments pave the way for the progress of hypopharyngeal carcinoma [16]. In this study, OS was statistically higher in postoperative RT patients (58.3% vs. 45.8%). However, it is necessary to take into consideration the bias induced by retrospective methods, for example, patients with poor conditions were often allocated to conservative treatments. There was a potential bias of age, marital status, body mass index, alcohol status, and economic situations. Additionally, our sample size might not be large enough to solve the bias caused by the tumor stage. Therefore, larger clinical trials should be designed to explore the feasibility and efficacy of therapeutic regimens.

### 4.1. Laryngeal Function Reconstruction Strategies

Preservation and maximization of laryngeal function is an important question. T_1–2_ postcricoid carcinoma is the best indication for laryngeal function preservation. Through intraoperative observation, we found that most T_3–4_ tumors may invade the inner wall of the pyriform sinus, cricoid cartilage, and cervical esophagus downward. When postcricoid carcinoma only invaded a small part of cricoid cartilage with no involvement in the contralateral larynx and esophageal entrance, part or total laryngeal function could be preserved in patients without obvious dysphagia. When postcricoid carcinoma infiltrated cricoid cartilage and laryngeal tissue extensively through the inner wall of bilateral pyriform sinus, it was unsafe to retain positive frozen section margins, extensive resection of the whole laryngeal and hypopharynx was necessary.

Some domestic surgeons believe that preserving laryngeal function improves survival outcomes for patients with hypopharyngeal carcinoma [17]. As a result of contradictory data, OS in this study was not statistically higher in patients who preserved laryngeal function, consistent with most scholars at home and abroad, which possibly be related to comparable baseline, disease characteristics, metastases, and even psychosocial consequences [18]. Moreover, laryngeal function preservation did not raise the risk of surgical-induced morbidities such as pharyngeal fistula, decannulation, and locoregional relapse (*p* > 0.05). The incidence of pharyngeal fistula was 25/72 (34.7%) in our cohort, which is similar to the incidence of other empirical studies ranging from 10% to 50% (sometimes increased up to 60%) in hypopharyngeal reconstruction, as reported in [19]. Notably, preoperative NAC and low T stage (tumor diameter ≤ 2 cm) particularly reduced pharyngeal fistula rate by chi-square test. Best of all, the median hospitalization time after pharyngeal fistula was not significantly longer and the total hospitalization did not cost much more than the others in our cohort. As to various surgical procedures, we found equivalent oncological results in swallowing function, in brief, 48/72 (66.7%) cases were free of nasogastric feeding after stage I operation, with a mean of 21.7 days.

The materials we used to reconstruct mucosal vacancy depended on the extent of pharyngeal, laryngeal, and esophageal defects. If the tumor range was limited, retaining mucosa, epiglottic flap, or sternohyoid myofascial flap could be used. If the defect range was extensive after resection, the sternohyoid myofascial flap, pectoralis myocutaneous flap, gastric transposition, colonic migration, or free jejunum could be used [20]. The functional larynx is defined as swallowing without aspiration, breathing without tracheostomy, with performing an understandable voice [21]. This study resulted in a satisfactory functional larynx with a preservation rate (50/72, 69.4%) and decannulation rate (18/50, 36%). After reconstruction of the phonatory tube, although 96% of patients developed slight and moderate hoarseness by subjective, 36% of patients met the standard of a functional larynx. Based on the aggressive characteristics of postcricoid carcinoma, reconstruction methods are challenging without correlative summaries reported. Our experience of reconstruction is as follows.

After the resection of the primary, the bilateral arytenoid cartilage is seldom reserved. The aryepiglottic fold and postcricoid mucosa of the affected side can be twisted backward to the posterior border of the ventricular cord, as close as possible to the tongue base so that the laryngeal inlet is slightly inclined downward, to reduce the morbidity rates of aspiration and pulmonary infection, which has some similarities to another large retrospective cohort study by Jin et al. [17].

As to the lateral wall margin of the laryngopharynx, the epiglottis can be pulled down or rotated outward and sutured with the edge of subglottic tissue. If the epiglottis is removed, a tongue flap could be utilized to cover the laryngeal inlet to avoid aspiration [22].

The pectoralis myocutaneous flap is suitable for non-circle defects like the sidewall defects, under the condition that the esophagus stump above the thoracic inlet can be anastomosed. However, the pectoralis myocutaneous flap is prone to developing pharyngeal fistula relatively, while the fundus of the stomach involves abundant blood supply to connect with pharyngeal mucosa. Additionally, we suggest that the gastric tube be placed on the affected side, which may maintain constant tension around the flap and prevent anastomotic stenosis to some extent.

If the lower edge of the tumor is low, gastric transposition or colonic migration can be used to restore the lower hypopharynx and esophagus. On the other hand, regarding the skip metastases of postcricoid carcinoma, removal of the whole esophagus eliminates occult metastases, and the safety margin is guaranteed as well.

Due to the protective anatomic effect of the laryngeal cartilaginous, the laryngotracheal flap is applied to patients who cannot tolerate gastric transposition or colonic migration. In our experience, one side of the thyroid superior arteriovenous should be preserved in case pharyngeal fistula develops in the lower anastomosis of the cervical trachea and esophagus.

Last but not least, pay attention to protecting recurrent laryngeal nerve during the operation.

### 4.2. Management of Cervical Lymph Node Metastasis

Recent studies have confirmed that a positive cervical lymph node is the most critical factor influencing survival in non-oropharyngeal head and neck carcinoma [23]. Similarly, lymph node metastasis was the only prognostic factor of morbidity by multivariate analysis. Moreover, 12 cases died of cervical metastasis and carotid artery invasion. It is reported that the recurrence rate of cervical lymph nodes in hypopharyngeal carcinoma was 17.5–27.3% [24], which appeared to be 37/72 (51.4%) in our study, which unveils a higher malignant degree of metastatic lymph nodes in postpricoid carcinoma.

Additionally, we investigated that neck recurrence did not benefit from RT; there were 28/48 (58.3%) irradiated patients with recurrence. This is possibly not only related to extended nodal presentation, but also to micrometastases in patients with cN0-1 postcricoid carcinoma. Nearly 40% of patients have been found with micro lymph node metastasis as reported, so the first station lymph node dissection is recommended in cN0 cases, especially in those without adjuvant radio (chemo)therapy in lymphatic drainage territory [25,26]. Lymph nodes have been identified as more radioresistant than the primaries, however, the efficacy of radiotherapy on patients with neck recurrence after neck dissection has been still disappointed in recent years [27].

Lymphatic vessels in the hypopharynx region are abundant, II–III regions are the first neck lymph node metastasis station, IV region is the second station, and I and V regions are the third [28]. The NCCN guidelines suggested that lymph node dissection should include ipsilateral II–IV regions [11]. In this study, neck dissection extent remained statistically insignificant in OS and DFS, which is consistent with that of Park et al. [25]. In advanced nodal disease, radical neck dissection sacrifices the accessory nerve, jugular vein, and sternocleidomastoid muscle, therefore, there is controversy regarding the disadvantages of radical surgery.

This study analyzed patients in a broad period, during which much has changed in terms of surgical instruments, equipment, and multimodal regimens over the years. For our statistics, neck recurrence frequently stagnates after almost 12 months; we advise attaching great importance to the first 2 years after the first neck dissection, including enhanced CT/MRI and positron emission tomography (PET). Jones et al. found that a radical neck resection after the first neck dissection could achieve a one-third cure rate [29]. Our data showed that nearly two-thirds of neck recurrence patients (67.6%) were freed from relapse after salvage surgery. Therefore, patients with neck recurrence should not give up salvage operations.

### 4.3. Multimodal Treatments

In addition to surgery, the intensification of adjuvant therapy is considered effective in improving oncological outcomes. It was proved by two corresponding stage III randomized trials (EORTC 24891, NCT00095875) in advanced hypopharyngeal carcinoma that NAC may provide more chances to preserve laryngeal function without reducing the local control rate and OS [30]. Subsequent clinical studies reached analogous conclusions, although NAC could not prolong OS, the effective rate increased from 10% to 26% [31]. Similarly, 17/33 (51.5%) cases achieved partial remission (PR) in this study; the laryngeal function preservation rate of the NAC group was 23/33 (66.7%), whereas OS was similar to those who did not receive NAC. Our data also found a lower rate of overall morbidity. Locoregional relapse was rarely observed in NAC patients (2/33, 6.1%); moreover, NAC showed absolute improvement in the development of pharyngeal fistula. It is reinforced that NAC is an important predictor of reducing morbidities.

As for patients with surgical contraindications (e.g., T4 postcricoid carcinoma, >70 years old or in poor nutritional status), multimodal nonsurgical treatment, preferably radiotherapy, could be a useful option for the reduction of locoregional control and adverse effects. Researchers reported that patients of III–IVB stage hypopharyngeal carcinoma gained a 5-year OS of 62% with definitive CRT [32]. Favorable laryngeal function preservation rate and oncological outcomes can be achieved utilizing IMRT, and our data confirmed the potential of IMRT as well. As we expected, the 3- and 5-year survival rates and OS were much better in RT populations, while insignificant outcomes were shown in the management of locoregional and lymph node relapses. Therefore, it is evident to find a balance between intensive treatment and surgery, more data are required to compare the efficacy of surgical-based treatment with radiotherapy-based non-surgical treatment. Metastatic lymph nodes are also of great significance to the prognosis and adjuvant treatment strategies, which were verified to benefit from CRT compared with RT alone [33]. Furthermore, the combination of monoclonal antibody panitumumab with postoperative RT improves progression-free survival (PFS) [34]. However, this work did not analyze the effects of RT and CRT patients, because there were few RT patients in association with cisplatin (less than 10), which may cause serious statistical deviation. Traditionally, oncologic outcomes focus mainly on the quantity rather than the quality of survival. Nowadays, oral nutrition, swallowing, voice rehabilitation, and verbal communication ability are vital for the quality of life (QoL) in patients with hypopharyngeal carcinoma [35]. Our surgical reconstruction provided satisfied swallowing and voice functional outcomes. Intriguingly, postoperative RT patients with partial laryngeal function had a lower rate of decannulation (8/33, 24.2%), which may be strongly associated with the advanced tumor stage and lymph node burden, to some extent, with increasing toxicity. However, the benefits of RT were probably abrogated by deviations in T and N stage, undergoing NAC or not, or inability to receive adequate doses (in particular the old and weak) in this cohort. More importantly, QoL was not assessed at the conclusion of surgery or RT when it was likely to be at its nadir.

The side effects of RT and NAC should not be neglected. Short-term NAC complications include oral mucositis, skin fibrosis, ulceration, and necrosis. Two of our patients suffered from a large skin rash on the back after the first course of chemotherapy. Long-term morbidities in RT include severe dysphagia, tissue ulceration, and palsy of the XI and XIIth cranial nerves. Under this condition, salvage surgery may encounter more difficulties due to tissue adhesion and necrosis of the covered area. Finally, this study has certain limitations. This cohort selection relied on surgical patients inherently, the willingness for definitive nonsurgical treatment may reflect residual biases, which are not fully captured in this analysis.

## 5. Conclusions

In recent years, surgery is regarded as the core part of multimodal treatments. Laryngeal function preserved surgical procedures are well managed in experienced otorhinolaryngology-head and neck surgery departments. In this study, we utilized sternocleidomastoid flap, epiglottis flap, platysma, and pectoralis musculocutaneous flap, gastric transposition, and colonic migration to reconstruct laryngeal and hypopharyngeal defection, combined with neoadjuvant chemotherapy and radiotherapy. Optimizing patients’ social adaptability after complete resection of the primary is a significant challenge in surgical treatment today. In this cohort, a poor prognosis was associated with low tumor differentiation, lymph node metastasis, tobacco consumption, and neck recurrence, while radiotherapy benefited survival. Meanwhile, lymph node metastasis was the most aggressive factor in cancer progression by multiple regression. NAC and tumor diameters ≤ 2 cm were confirmed to be protective factors of the occurrence of pharyngeal fistula and locoregional relapse. Nowadays, multimodal treatment efforts like NAC and RT are made concerning improving survival and reducing postoperative complications under an interprofessional model with clinical oncologists. However, the indications and timing of an appropriate therapeutic strategy are not a simple problem, more studies especially prospective studies should be conducted to support the fundamental principles of postcricoid carcinoma treatment.

## Figures and Tables

**Table 1:** TNM stages of postcricoid carcinoma patients.

Stage	Laryngeal Function Preserved (*n =* 50)				Laryngeal Function Not Preserved (*n =* 22)			
	T_1_	T_2_	T_3_	T_4_	T_1_	T_2_	T_3_	T_4_
N_0_	5	5	0	7	1	2	0	4
N_1_	3	0	1	7	3	1	1	3
N_2_	4	5	4	8	0	2	1	3
N_3_	0	0	0	1	0	0	0	1
Total	12	10	5	23	4	5	2	11

**Table 2:** Treatment and clinical characteristics of patients.

Value	Number (*n =* 72)	Survival		χ2	*p*-Value
		Live	Death		
(a) TNM stage					
I–II stage	13	8	5	0.04	0.85
III–IV stage	59	28	31		
(b) Larynx preservation					
Yes	50	24	26	0.05	0.83
No	22	12	10		
(c) Neck dissection					
Radical neck dissection	21	11	10	1.50	0.22
Elective neck dissection	51	25	26		
(d) Neoadjuvant chemotherapy					
Yes	33	17	16	0.05	0.81
No	39	19	20		
(e) Tumor differentiation					
High/medium	29	23	6	7.89	0.01
Low	43	13	30		
(f) Lymph node metastasis					
cN0	24	18	6	7.5	0.01
cN+	48	18	30		
(g) Tumor size					
diameter > 2 cm	56	28	28	0.23	0.63
diameter ≤ 2 cm	16	8	8		
(h) Pharyngeal fistula					
Yes	25	9	16	1.09	0.29
No	47	27	20		
(i) Locoregional relapse					
Yes	39	21	18	0.79	0.37
No	33	15	18		
(j) Smoke history					
Yes	36	16	20	16.12	< 0.001
No	36	20	16		
(k) Postoperative radiotherapy					
Yes	48	26	22	4.01	0.04
No	24	10	14		
(l) Neck recurrence					
Yes	37	6	31	41.13	< 0.001
No	35	30	5		

**Table 3:** Multivariate Cox analysis of treatment and clinical characteristics.

Value	*p* Value	Hazard Ratio (95% CI)	Forest Plot
Tumor differentiation	0.01	0.29 (0.11-0.72)	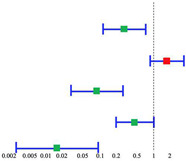
Lymph node metastasis	0.13	1.75 (0.85–3.59)	
Smoke history	0.00	0.09 (0.03–0.27)	
Postoperative radio-therapy	0.05	0.44 (0.19–1.00)	
KPS score	0.00	0.02 (0.00–0.09)	

**Table 4:** Treatment and clinical characteristics related to pharyngeal fistula.

Group		Pharyngeal Fistula (*n =* 25)	No Pharyngeal Fistula (*n =* 47)		*p*-Value
Larynx preservation	Yes	16	34		0.47
	No	9	13		
Neoadjuvant chemotherapy	Yes	0	33		<0.001
	No	25	14		
Postoperative radiotherapy	Yes	14	34		0.16
	No	11	13		
Tumor size	*>* 2 cm	23	33		0.03
	≤2 cm	2	14		
TNM stage	I–II stage	5	8		0.75
	III–IV stage	20	39		
Neck dissection	Radical	7	14	0.87	
	Elective	18	33		

**Table 5:** Treatment and clinical characteristics related to decannulation.

Group		Decannulation (*n =* 18)	Without Decannulation (*n =* 32)	*p*-Value	
Neoadjuvant chemotherapy	Yes	6	17	0.18	
	No	12	15		
Postoperative radiotherapy	Yes	8	25	0.02	
	No	10	7		
Tumor size	>2 cm	12	24	0.53	
	≤2 cm	6	8		
TNM stage	I–II stage	4	6	0.77	
	III–IV stage	14	26		
Pharyngeal fistula	Yes	8	8		0.16
	No	10	24		

**Table 6:** Treatment and clinical characteristics related to locoregional relapse.

Group		Locoregional Relapse (*n =* 39)	No locoregional Relapse (*n =* 33)	*p*-Value
Neoadjuvant chemotherapy	Yes	2	31	<0.001
	No	37	2	
Postoperative radiotherapy	Yes	24	24	0.32
	No	15	9	
Tumor size	>2 cm	37	19	<0.001
	≤2 cm	2	14	
TNM stage	I–II stage	6	7	0.52
	III–IV stage	33	26	
Neck dissection	Radical	12	9	0.75
	Elective	27	24	

**Table 7:** Treatment and clinical characteristics related to neck recurrence.

Group		Neck Recurrence (*n =* 37)	No neck Recurrence (*n =* 35)	*p*-Value
Lymph node metastasis	cN_0_	8	16	0.03
	cN_+_	29	19	
Neoadjuvant chemotherapy	Yes	19	14	0.33
	No	18	21	
Postoperative radiotherapy	Yes	28	20	0.10
	No	9	15	
Tumor size	>2 cm	27	29	0.31
	≤2 cm	10	6	
TNM stage	I–II stage	8	5	0.42
	III–IV stage	29	30	
Neck dissection	Radical	10	11	0.68
	Elective	27	24	

## Data Availability

The data presented in this study are available on request from the corresponding authors.
